# Police-referred psychiatric emergency presentations during the first and second wave of COVID-19 in Berlin, Germany: a retrospective chart review

**DOI:** 10.1186/s12888-024-05903-z

**Published:** 2024-06-12

**Authors:** Thomas Goldschmidt, Yann David Kippe, Stefan Gutwinski, Karl Deutscher, Meryam Schouler-Ocak, Franziska Kroehn-Liedtke

**Affiliations:** 1 Psychiatrische Universitätsklinik der Charité im St. Hedwig Krankenhaus, Große Hamburger Str. 5-11, Berlin, Germany; 2 Amsterdam University Medical Center, Amsterdam, The Netherlands

**Keywords:** Police, COVID-19, Psychiatric emergency, Schizophrenia

## Abstract

**Background:**

Literature on psychiatric emergency services (PES) presentations during the COVID-19 pandemic showed heterogeneous results regarding patients brought in by police (BIBP). This is the first study primarily focusing on patients BIBP in a PES during the COVID-19-period.

**Methods:**

Case documentation records during the first and second wave of the COVID-19 pandemic in a PES in Berlin, Germany were analyzed using descriptive data analysis and binomial logistic regression analysis to detect factors that predict presentations BIBP.

**Results:**

5440 PES presentations: 20.4% BIBP during the first wave vs. 16.3% during its control period; second wave: 17.6% BIBP vs. 14.9% during its control period. In both waves, absolute increases in presentations BIBP were seen compared to control (*p* = .029, *p* = .028, respectively). COVID-19-period was a predictor for presentations BIBP during the first and the second wave. The following factors also predicted presentations BIBP: younger age, male gender, aggressive behavior, suicide attempt prior to presentation and diagnosis of psychotic or substance use disorders; depressive disorders were negatively associated.

**Conclusions:**

During the two first waves of the COVID-19 pandemic, there was an increase in presentations BIBP in a PES in Berlin. Regression analysis shows that the pandemic itself was a predictor of presentations BIBP. The underlying factors of this association need to be further elucidated in future research. Additionally, general factors predicting PES presentations BIBP are reported that replenish the present literature.

## Background

Police plays a crucial role in referring individuals to emergency services [1, 2]. It is the police that makes the initial decision whether to refer persons with apparent or assumed mental health problems to the justice system or rather to health care services. The proportion of patients brought in by police (BIBP) to general emergency services ranges around 0.8–1.3% according to a recent review [1]. The group of patients BIBP has high rates of mental health problems with frequent substance use disorders and a high proportion of young (median age in the early 30 s) males [1]. Often, however, police directly transfers patients to specialized psychiatric emergency services (PES). Studies from Australia, Taiwan and the US report a proportion from 9.1–39.6% of patients BIBP in PES presentations [3–7].

There is evidence suggesting that differences in police training (such as the implementation of crisis intervention teams) may influence the rate of patients BIBP [8]. Other factors that may influence the rate of patients BIBP to PES are local-bound differences regarding the sociodemographic and clinical characteristics [6, 9]. Studies on presentations BIBP in PES report mostly a male preponderance, a history of violence and violence or threat of violence towards others directly prior to presentation [3], a longer time in the emergency ward [3], and some studies suggest that patients BIBP are more often admitted as inpatients than others [4, 6]. Results on differences regarding diagnostic groups are inconsistent. Sales and Way et al. state that there were no differences in psychiatric diagnostic groups between patients BIBP (*N* = 62 and *N* = 107, respectively) and those referred by other sources (*N* = 283 and *N* = 255, respectively) [5, 6]. Redondo et al. (*N* = 100 vs *N* = 279) found a higher amount of severe psychosocial stressors in the group of patients BIBP [3]. Wang et al. (*N* = 3029 vs. *N* = 7656) found substance use disorders and a diagnosis of unspecified psychosis more often in the group of patients BIBP [4].

The outbreak of the Coronavirus Disease (COVID-19) in late 2019 brought unprecedented challenges to health care systems worldwide, affecting various aspects of people's lives with direct somatic health consequences and direct and indirect repercussions on mental health as exacerbating pre-existing mental illnesses [10]. Since the declaration of COVID-19 as a pandemic in March of 2020 by the WHO, its impact on mental health has been explored in various studies, suggesting an increase in the prevalence of psychiatric disorders e.g. depression, anxiety and substance use [11–13].

In the beginning of the COVID-19 pandemic, a decrease in psychiatric emergency presentations was observed worldwide, ranging from 4% [14] to 56% [15]. Possible reasons hypothesized to explain the decrease are the fear of infection, social distancing measures or news about limited capacities in emergency facilities [16–20].

Regarding PES presentations BIBP, findings were heterogeneous: some studies reported increases of patients BIBP to PESs during the pandemic [9, 21]; others found a decrease in PES presentations BIBP [22, 23]. However, no studies until date, primarily focused on the group of patients BIBP to PES during the COVID-19 pandemic.

On the background of heterogeneous and scarce evidence regarding presentations BIBP to PES, this study aims to contribute to a more elaborate picture of this group of psychiatric patients during the COVID-19 pandemic. The primary goal of this exploratory study is to identify predictive factors for PES presentations BIBP during the COVID-19 pandemic.

## Methods

### Study design

A retrospective chart review of case documentation records of presentations to the PES of Charité Berlin at St. Hedwig Hospital (SHK) was conducted during the first and second wave of the COVID-19 pandemic in Germany in this monocentric study. Local ethics committee (Charité University, Berlin: EA110/20) approved the study. The first wave was considered the time period from 03/02/2020 with the first publicized case of COVID-19 in Berlin until 05/24/2020 with the infection curve reaching its bottom number of newly registered cases of infections [24]. The second wave was defined as the period between 09/15/2020, being characterized by a continuous increase of 7-day incidences and 03/01/2021, the date when lockdown measures were firstly rescinded in Berlin [25]. As comparison, we defined the same period one year earlier as the control period. The period in between the two waves was characterized by very low infection rates and social life was almost back to normal again. Hence, this in between-period was omitted from the study.

The psychiatric department at SHK comprises an emergency department and seven psychiatric care units for inpatient treatment. As a district hospital it supplies the districts of Berlin Moabit, Tiergarten and Wedding, a catchment area of approx. 327.000 people. The central railway station is also part of this area and often a source of police referral of psychiatric patients from all over Germany.

PES presentations were analyzed on patient-level with clinical characteristics (diagnoses, suicidal thoughts, suicide attempts) and socio-demographic data (age, gender, living status) and on the event-level that provides information on the absolute number of PES presentations during the different periods.

As in earlier publications on this cohort [9, 13, 26], cases were excluded if they concerned scheduled admissions to psychiatric wards or admissions to a day therapy unit. The latter were shut down during the beginning of the pandemic. Additional exclusion criteria were: patients who left without being seen by a psychiatrist or when no psychiatric F-diagnosis according to the International Statistical Classification of Diseases and Related Health Problems, 10th revision (ICD-10) was documented.

Considering high frequent attenders and the possible bias this group would impose on our results, we merged inpatient stays if they were separated by less than 3 days. If cases were separated by 4–7 days, they were only merged if discharge was because of somatic complications or against documented advice of medical staff.

For analysis of our data, we grouped cases into 4 different diagnostic categories based on the principal diagnosis: substance use disorders (ICD-10 diagnoses F10-F19 except for nicotine related disorders (F17) and substance related psychotic disorders (F1x.5 and F1x.7), schizophrenia and psychotic disorders (F20-F29), bipolar and manic disorders (F30-F31), depressive disorders (F32-F33). As the analysis regarding the secondary outcome (description of the group of patients BIBP independently of COVID-19) was of a more exploratory nature, we added to this analysis two more diagnostic groups: organic mental disorders (F00-F09) and personality disorders (F60-F62).

### Statistical analysis

Quantitative variables were tested for normal distribution utilizing the Kolmogorov–Smirnov-Test and via graphic examination of the Q-Q-Plot. Since all quantitative variables were not normally distributed, only medians are reported; for description of qualitative variables, absolute numbers and percentages are presented. Comparisons of medians between groups were performed using the Mann–Whitney-U-Test, comparisons of percentages between groups were performed using the Chi^2^ test unless otherwise stated. For statistical tests where expected case numbers were lower than 5, the Fisher-exact-test was used. The *p*-value for statistical significance was set to *p* < 0.05 except for the testing of 8 potential predicting variables for presentations BIBP in Tables 1 and 2. For these analyses, we applied a Bonferroni correction for multiple testing as follows: *p* = 0.05/8 = 0.0063.

**Table 1:** Clinical and demographic characteristics of PES presentations BIBP and not BIBP during the first wave and its control period

	First Wave not BIBP	First Wave BIBP	*p*-value	chi^2^	Control period of First Wave not BIBP	Control period of First Wave BIBP	*p*-value	chi^2^
*N* total number of cases	647	166	-		748	146	-	
Mean cases per week	53.92	13.83	** < .001**	T: 14.617	62.33	12.17	** < .001**	T: 19.448
Mean age	43.08y (15.91)	38.83 y (14.20)	**.001**	T: 3.355	41.55y (16.04)	38.81 y (15.71)	.059	T: 1.892
*N* females	244 (37.7%)	67 (40.4%)	.531	0.392	345 (46.1%)	38 (26.0%)	** < .001**	20.145
Tested positive for COVID-19	0	0	-		-	-	-	
Inpatient admission	260 (40.2%)	103 (62.0%)	** < .001**	25.55	331 (44.3%)	106 (72.6%)	** < .001**	39.296
Involuntary admission	21 (3.2%)	46 (27.7%)	** < .001**	104.6	24 (3.2%)	55 (37.7%)	** < .001**	180.1
*Living alone*	192 (29.7%)	47 (28.3%)	.731	0.118	188 (25.1%)	33 (22.6%)	.517	0.42
*Aggressive behavior towards others*	27 (4.2%)	60 (36.1%)	** < .001**	139.8	30 (4.0%)	72 (49.3%)	** < .001**	248.05
*Suicidal thoughts*	181 (29.0%)	34 (23.4%)	.183	1.777	171 (24.7%)	24 (19.4%)	.195	1.679
*Suicide attempts*	25 (3.9%)	12 (7.5%)	.053	3.729	18 (2.4%)	2 (1.4%)	.455	0.558
*Substance use disorders*	202 (31.2%)	51 (30.7%)	.902	0.015	224 (29.9%)	58 (39.7%)	.020	5.41
*Schizophrenia spectrum/psychotic disorders*	153 (23.6%)	59 (35.5%)	**.002**	9.696	168 (22.5%)	40 (27.4%)	.197	1.668
*Bipolar manic disorders*	36 (5.6%)	14 (8.4%)	.170	1.885	32 (4.3%)	9 (6.2%)	.319	0.993
*Depressive disorders*	77 (11.9%)	3 (1.8%)	** < .001**	15.17	98 (13.1%)	3 (2.1%)	** < .001**	14.875

Comparison of demographic and clinical characteristics of PES presentations with and without police; *p*-values (bold = significant to a level of *p* ≤ .05 or *p* ≤ .0063 after Bonferroni correction for potential predictive variables for BIBP = in italic font) are derived from chi^2^- tests unless differently stated (T = Student's T test). All chi^2^- tests with df = 1 except for statistical test on COVID-19 positive: df = 6. "Covid-19 positive" includes all patients tested at admission or during hospital treatment

**Table 2:** Clinical and demographic characteristics of pES presentations BIBP and not BIBP during the Second Wave and its control period

	Second Wave not BIBP	Second Wave BIBP	*p*-value	chi^2^	Control period of Second Wave not BIBP	Control period of Second Wave BIBP	*p*-value	chi^2^
N total number of cases	1566	334	-	-	1578	277	-	-
Mean cases per week	65.25	13.92	** < .001**	T: 30.591	65.75	11.54	** < .001**	T: 37.549
Mean age	42.59 y (16.47)	38.72 y (14.43)	**.001**	T: 3.217	42.81 y (16.34)	38.92 y (13.34)	** < .001**	T: 4.316
N females	647 (41.3%)	109 (32.6%)	**.003**	8.709	657 (41.6%)	75 (27.1%)	** < .001**	20.908
Tested positive for COVID-19	6 (0.4%)	0 (0.0%)	.107	10.435	-	-	-	-
Inpatient admission	620 (39.6%)	189 (56.6%)	** < .001**	32.418	647 (42.7%)	186 (67.1%)	** < .001**	56.58
Involuntary admission	69 (4.4%)	116 (34.7%)	** < .001**	287.81	66 (4.2%)	93 (33.6%)	** < .001**	259.75
*Living alone*	524 (33.5%)	90 (26.9%)	.020	5.375	322 (20.4%)	50 (18.1%)	.367	0.815
*Aggressive behavior towards others*	52 (3.3%)	131 (39.2%)	** < .001**	405.65	87 (5.5%)	119 (43.4%)	** < .001**	338.51
*Suicidal thoughts*	388 (24.9%)	79 (23.9%)	.703	0.145	411 (27.0%)	72 (28.9%)	.530	0.394
*Suicide attempts*	54 (3.5%)	29 (8.7%)	** < .001**	18.175	48 (3.1%)	29 (10.6%)	** < .001**	33.065
*Substance use disorders*	469 (30.0%)	133 (39.8%)	** < .001**	12.34	448 (28.4%)	112 (40.4%)	** < .001**	16.216
*Schizophrenia spectrum/psychotic disorders*	343 (21.9%)	97 (29.0%)	**.005**	7.85	347 (22.0%)	82 (29.6%)	**0.006**	7.682
*Bipolar manic disorders*	73 (4.7%)	10 (3.0%)	.175	1.838	56 (3.5%)	25 (9.0%)	** < .001**	16.924
*Depressive disorders*	193 (12.3%)	10 (3.0%)	** < .001**	25.142	207 (13.1%)	5 (1.8%)	** < .001**	29.792

Comparison of demographic and clinical characteristics of PES presentations with and without police; *p*-values (bold = significant to a level of *p* ≤ .05 or *p* ≤ .0063 after Bonferroni correction for potential predictive variables for BIBP = in italic font) are derived from chi^2^- tests unless differently stated (T = Student's T test). All chi^2^- tests with df = 1 except for statistical test on COVID-19 positive: df = 6. "Covid-19 positive" includes all patients tested at admission or during hospital treatment

We examined risk factors for PES presentations BIBP by means of hierarchical binominal logistic regression in each observed COVID-19-period with its corresponding control-period. As predictor variables, we first entered (block 1) sociodemographic features (age, female gender, living alone), then (block 2) psychopathological features (aggressive behavior towards others, suicidal thoughts, suicide attempt prior to PES presentation) and diagnostic subgroups (substance use disorder, schizophrenia spectrum/psychotic disorders, bipolar manic disorders, depressive disorders). Subsequently (block 3), we entered “COVID-19-period” and interactions with COVID-19-period (block 4). The above mentioned predictor variables were only included in the regression under the condition that they proved to be statistical significant in the descriptive statistics (Tables 1 and 2). Results of the hierarchical binomial logistic regression models are displayed as Odd’s Ratios (Exp(B)) with 95% confidence intervals (95%CI). The overall level of significance was set to *p* < 0.05. Problems regarding collinearity were ruled out by means of standard statistical procedures (comparison of correlation matrices).

## Results

**Fig. 1:**
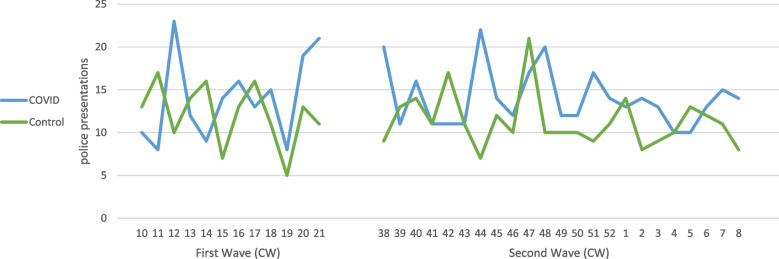
PES presentations with police per week for both waves and control periods (CW=calendar weeks)

During the two COVID-19 time periods and the respective control periods, a total of 5440 psychiatric emergency service (PES) presentations were included in this study. During the first wave, 20.4% of PES presentations (166 out of 813) were BIBP, compared to 16.3% (146 out of 894) during the control period. As to the second wave, which was twice as long as the first wave, 17.6% of PES presentations were BIBP (334 out of 1566), compared to 14.9% (277 out of 1578) during the second wave’s control period. The higher percentages of presentations BIBP during the COVID-19 periods as compared to the control periods reached statistical significance for both waves (first wave: *p* = 0.029, second wave: *p* = 0.028). Figure 1 illustrates the PES presentations BIBP per week for both waves and control periods.

Table 1 shows the clinical and demographic characteristics of PES presentations BIBP and not BIBP during the first wave and during its control period, respectively.

Table 2 shows the clinical and demographic characteristics of PES presentations BIBP and not BIBP during the second wave and during its control period, respectively.

Table 3 shows the results of the binomial logistic regression analysis. As to the first wave and its control period, the following variables predicted the outcome: patients BIBP, positively: COVID-19-period (Exp(B) = 1.435, *p* = 0.033), diagnosis of schizophrenia spectrum/psychotic disorders (Exp(B) = 1.614, *p* = 0.010) and aggressive behavior towards others (Exp(B) = 20.022, *p* < 0.001). Age and diagnosis of depressive disorders predicted the outcome: patients BIBP, negatively (Exp(B) = 0.985, *p* = 0.008and Exp(B) = 0.275, *p* = 0.003, respectively). All other variables (female gender, living alone, suicidal thoughts, suicide attempts, substance use disorders, and bipolar manic disorders) did not predict the outcome: patients BIBP during the first wave and its control period. Entry of interaction effects with COVID-19-period did not improve the model (Table 4).

**Table 3:** Binominal hierarchical Regression Analysis, outcome: patients BIBP

	First wave + control period				Second wave + control period			
	Exp (B)	95% CI lower	95% CI upper	*p*-value	Exp (B)	95% CI lower	95% CI upper	*p*-value
**COVID-19-period**	1.435	1.029	2.003	**0.033**	1.458	1.146	1.854	**0.002**
**Age**	0.985	0.975	0.996	**0.008**	0.982	0.974	0.989	** < .001**
**Female gender**	0.885	0.625	1.254	0.493	0.697	0.539	0.903	**0.006**
**Aggressive behaviour towards others**	20.022	12.962	30.930	** < .001**	14.477	10.650	19.679	** < .001**
**Suicide attempts**	-	-	-	-	5.194	3.354	8.042	** < .001**
**Substance use disorders**	-	-	-	-	1.701	1.246	2.323	**.001**
**Schizophrenia spectrum/psychotic disorders**	1.614	1.119	2.327	**0.010**	2.010	1.457	2.772	** < .001**
**Bipolar manic disorders**	-	-	-	-	1.625	0.928	2.844	0.089
**Depressive disorders**	0.275	0.117	0.647	**0.003**	0.376	0.204	0.692	**0.002**

Results of hierarchical logistic regression analysis on factors potentially associated with presentation with police. Bold print indicates statistical significance at *p* ≤ .05 level. Variables were only included when significant in Tables 1 or 2

**Table 4:** Adding interaction effects with COVID-19-period does not improve the model

**Block 3 (adding COVID-19-period to the model)**	**First wave + control period**			**Second wave + control period**		
	***chi***^***2***^	***df***	***p-value***	***chi***^***2***^	***df***	***p-value***
**Step**	4.558	1	**0.033**	9.497	1	**0.002**
**Block**	4.558	1	**0.033**	9.497	1	**0.002**
**Model**	278.482	6	**0.000**	488.591	10	**0.000**
**Block 4 (adding interaction effects with COVID-19- period to the model)**	**First wave + control period**			**Second wave + control period**		
	***chi***^***2***^	***df***	***p-value***	***chi***^***2***^	***df***	***p-value***
**Step**	4.958	5	0.421	10.842	9	0.287
**Block**	4.958	5	0.421	10.842	9	0.287
**Model**	283.441	11	**0.000**	499.433	19	**0.000**

Omnibus tests of entering block 3 and block 4 to the hierarchical regression models. *P*-values of step and block after entering block 3 are statistically significant, indicating that the variable COVID-19 period adds to the explanation of variance. *P*-values of step and block after entering block 4 are not statistically significant, indicating that interactions with COVID-19-period do not add to the explanation of variance. df = degrees of freedom. Bold print indicates statistical significance at *p* ≤ .05 level

Table 3 also depicts the Regression analysis results regarding the second wave and its control period. The following variables predicted the outcome: patients BIBP during these periods, positively: COVID-19-period (Exp(B) = 1.458, *p* < 0.002), schizophrenia spectrum and psychotic disorders (Exp(B) = 2.010, *p* < 0.001), aggressive behavior towards others (Exp(B) = 14.477, *p* < 0.001), suicide attempts (Exp(B) = 5.194, *p* < 0.001) and substance use disorders (Exp(B) = 1.701, *p* = 0.001). Higher age, female gender and depressive disorders predicted the outcome: patients BIBP, negatively (Exp(B) = 0.982, *p* < 0.001, Exp(B) = 0.697, *p* = 0.006 and Exp(B) = 0.376, *p* = 0.002, respectively). All other variables (living alone and suicidal thoughts) did not predict the outcome: patients BIBP during the second wave and its control period. Entry of interaction effects with COVID-19-period did not improve the model (Table 4).

Figure 1 illustrates the comparison of weekly PES presentations referred by the police during the first and second wave of COVID-19 (blue lines) and their control periods (green lines).

## Discussion

This study is the first focusing primarily on PES presentations BIBP during the COVID-19 pandemic. The current study is also the first showing an absolute increase in PES presentations BIBP during the second wave compared to a control period one year earlier. We have shown the same for the first wave (based on a part of the current study’s sample [9]. What is more, this is the first study showing that COVID-19-period itself was a predictor for PES presentations BIBP, during the first and during the second wave compared to their control periods (Table 3).

One study from Taiwan on PES presentations during the COVID-19 pandemic did show an increase in police/emergency medical service presentations in 2021 but not in 2020, compared to pre-pandemic times [21]. Unfortunately, police and emergency medical service referrals were not reported separately which makes it difficult to compare to our study. Studies from Switzerland, Turkey and Australia cover only the first wave of the COVID-19 pandemic and report, in comparison to the current study, considerably lower rates of presentations BIBP and only minor changes to pre-pandemic times [22, 23, 27]. The current study’s rates are more in line with pre-pandemic studies [3–7]. One may assume that rates of presentations BIBP may differ between PES in more urban areas and those in more rural areas (although there is no scientific evidence for this assumption). When comparing the mentioned and the current study, however, all studies concern metropolitan areas. Furthermore, the differences in rates of presentations BIBP in PES in between different sites and countries are rather important. These differences may be due to country-bound differences such as different mental health care policies and police responsibilities on the one hand [8] and due to local-bound differences such as sociodemographic differences and differences in clinical characteristics on the other hand [4, 9]. More research is necessary to better understand the factors of influence of presentations BIBP to PES.

In the logistic regression analysis, COVID-19-associated effects were seen during the first and the second wave with almost identical odd’s ratios (1.435 and 1.458, respectively). These findings suggest that both waves similarly increased the probability of presentations BIBP to a PES in Berlin. As the entry of interaction effects (i.e. for ex. interactions between the presence of a specific psychiatric diagnosis and COVID-19-period) did not further add to improve the regression models (Table 4), we are not able to pinpoint a specific patient characteristic explaining the increase of presentations BIBP during the COVID-19 waves. This suggests that the COVID-19-period effect on presentations BIBP that we saw in our sample is rather complex and not mono-causal. Potentially, the explanatory factors may also differ between the two observed waves.

In comparison to other diagnostic groups, patients with schizophrenia spectrum/psychotic disorders are more likely to be BIBP (Table 3). Especially during the first wave, outpatient facilities were less available [28–32] with limited accessibility of many psychosocial [28] and psychotherapeutic [33] facilities. One may hypothesize that patients with chronic psychotic disorders and high need of psychosocial facilities might have suffered particularly from these constraints with exacerbation as a consequence. This view is supported by the fact that many studies show an increase in PES presentations of patients with psychotic disorders during the pandemic [16, 34–38].

In Tables 1 and 2 one can appreciate that inpatient admission and involuntary admissions are highly associated with patients BIBP, a finding that is highly plausible and has been reported earlier [39].

Independently of COVID-19, the following factors predicted presentations BIBP in all observation periods of our study: lower age, aggressive behavior towards others, and schizophrenia sprectrum/psychotic disorders(cf Table 3). Patients with depressive disorders were less likely to be BIBP. In the rather underpowered American studies (ca. 100 patients BIBP per study), age was not shown to be a predictor of presentations BIBP [6]. In more large-scale studies, such as Wang et al. from Taiwan (> 3000 patients BIBP), however, the group of patients between 30 and 39 years old were the most at risk of being BIBP [4]. This is in line with our findings. Aggressive behavior towards others has also been shown several times to be associated with presentations BIBP [3–6]. Psychotic disorders have earlier been reported in two small studies as potentially associated with police referrals [5, 40]. During the second wave and its control period, the presence of a substance use disorder, a suicide attempt prior to the presentation and male gender are predictors of patients BIBP. Both, the higher risk of being BIBP in patients with substance use disorder and the positive association of patients BIBP to a PES with suicide attempts prior to the presentation, are findings that were earlier reported in Taiwan [4] but not yet in Western countries. As depressive patients do rather often present with suicidal thoughts and after suicide attempts, the coincidental negative association with depressive disorders in patients BIBP might seem in the first place contradictory. However, these findings are in line with results of a meta-analysis conducted by Walker et al. in 2021 when we equalize BIBP and involuntary admission. They found that young patients with a primary diagnosis of affective disorders were significantly less at risk of involuntary admission when compared to patients perceived to be at risk of self-harm (including suicidal ideation or suicide attempts) (OR 2.05, *p* = 0.015) [41]. The same meta-analysis shows that young patients with substance use disorder were more likely to be admitted involuntarily (OR 1.87, *p* = 0.032) as well as patients who showed behavior of harm to others (e.g. aggression, violent acts) (OR 2.37, *p* = 0.002), which is also in line with the current study’s findings.

Male gender as predictor for presentations BIBP is a common finding in the literature [3–7].

### Strengths and limitations

This study is the first focusing primarily on PES presentations BIBP during the COVID-19 pandemic. The current study covers a relatively long observation period with a comparably large number of assessed PES presentations. Indicators of mental health were based on clinical diagnoses rather than self-reports. In addition, we performed a detailed clinician-led review of each case, based on thorough clinical documentation.

The following limitations need to be considered: the control data is limited to the previous year only. The study is based on clinical routine data which can differ in quality and extent which may introduce bias. We cannot completely rule out the possibility of an interrater bias. However, to limit this bias we implemented the following measures: consulting all available data and scheduling regular meetings to discuss pressing questions, resolving them in consensus.

A further limitation is that we only gathered information about patients BIBP in a single-center psychiatric emergency department. Extrapolation of results should therefore be done with caution.

## Conclusion

During the two first waves of the COVID-19 pandemic, there was an increase in presentations BIBP in a PES in Berlin. This is the first study showing that the COVID-19-period was a predictor for PES presentations BIBP during the first and the second wave. Factors such as reduced outpatient services and patient characteristics potentially played crucial roles. Understanding these dynamics is essential for healthcare systems to better prepare for and address the needs of individuals with psychiatric emergencies, both during and beyond public health crises. Thus, the complex interaction between the COVID-19 pandemic and PES presentations involving the police should be purpose of further research.

This is the most large-scale Western study yet on clinical factors predicting PES presentations BIBP. Factors that were found to predict presentations BIBP were: lower age, male gender, aggressive behaviour towards others, suicide attempts prior to presentation and diagnosis of schizophrenia spectrum and psychotic disorders or substance use disorders. Conversely, patients with depressive disorders were less likely to be BIBP. These findings replenish the presently scarce literature on patients BIBP to PES.

## Data Availability

The datasets used and/or analysed during the current study are available from the corresponding author on reasonable request.

## Abbreviations

BIBP: Brought in by police
PES: Psychiatric emergency service
COVID-19: Coronavirus disease caused by SARS-CoV-2
Exp(B): Odds ratio
CI: Confidence interval
